# Synergistic Antimicrobial Activity of Magnetite and Vancomycin-Loaded Mesoporous Silica Embedded in Alginate Films

**DOI:** 10.3390/gels9040295

**Published:** 2023-04-02

**Authors:** Georgiana Dolete, Cornelia-Ioana Ilie, Cristina Chircov, Bogdan Purcăreanu, Ludmila Motelica, Alina Moroșan, Ovidiu Cristian Oprea, Denisa Ficai, Ecaterina Andronescu, Lia-Mara Dițu

**Affiliations:** 1 Department of Science and Engineering of Oxide Materials and Nanomaterials, Faculty of Chemical Engineering and Biotechnologies, University Politehnica of Bucharest, Gh. Polizu 1-7, 011061 Bucharest, Romania; dolete.georgiana@gmail.com (G.D.); cornelia_ioana.ilie@upb.ro (C.-I.I.); cristina.chircov@yahoo.com (C.C.); bogdanpb89@gmail.com (B.P.); motelica_ludmila@yahoo.com (L.M.); denisaficai@yahoo.ro (D.F.); 2 National Center for Micro and Nanomaterials, University Politehnica of Bucharest, Splaiul Independenței 313, 060042 Bucharest, Romania; ovidiu73@yahoo.com; 3 BIOTEHNOS SA, Gorunului Street 3-5, 075100 Otopeni, Romania; 4 Department of Organic Chemistry “Costin Nenițescu”, Faculty of Chemical Engineering and Biotechnologies, University Politehnica of Bucharest, Gh. Polizu 1-7, 011061 Bucharest, Romania; ally_morosan@yahoo.com; 5 Department of Inorganic Chemistry, Physical Chemistry, and Electrochemistry, Faculty of Chemical Engineering and Biotechnologies, University Politehnica of Bucharest, Gh. Polizu 1-7, 011061 Bucharest, Romania; 6 Academy of Romanian Scientists, Ilfov Street 3, 050044 Bucharest, Romania; 7 Department of Microbiology and Immunology, Faculty of Biology, University of Bucharest, 1-3 Aleea Portocalelor, 060101 Bucharest, Romania; lia-mara.ditu@bio.unibuc.ro; 8 Research Institute of the University of Bucharest, 91-95 Splaiul Independenței, 050095 Bucharest, Romania

**Keywords:** antimicrobial wound dressings, hybrid nanostructured alginate films, synergistic activity, dual release mechanism, mesoporous silica, magnetite

## Abstract

The aim of the present study was to obtain a hydrogel-based film as a carrier for the sustained and controlled release of vancomycin, an antibiotic commonly used in various types of infections. Considering the high-water solubility of vancomycin (>50 mg/mL) and the aqueous medium underlying the exudates, a prolonged release of vancomycin from an MCM-41 carrier was sought. The present work focused on the synthesis of malic acid coated magnetite (Fe_3_O_4_/malic) by co-precipitation, synthesis of MCM-41 by a sol-gel method and loading of MCM-41 with vancomycin, and their use in alginate films for wound dressing. The nanoparticles obtained were physically mixed and embedded in the alginate gel. Prior to incorporation, the nanoparticles were characterized by XRD, FT-IR and FT-Raman spectroscopy, TGA-DSC and DLS. The films were prepared by a simple casting method and were further cross-linked and examined for possible heterogeneities by means of FT-IR microscopy and SEM. The degree of swelling and the water vapor transmission rate were determined, considering their potential use as wound dressings. The obtained films show morpho-structural homogeneity, sustained release over 48 h and a strong synergistic enhancement of the antimicrobial activity as a consequence of the hybrid nature of these films. The antimicrobial efficacy was tested against *S. aureus*, two strains of *E. faecalis* (including vancomycin-resistant Enterococcus, VRE) and *C. albicans*. The incorporation of magnetite was also considered as an external triggering component in case the films were used as a magneto-responsive smart dressing to stimulate vancomycin diffusion.

## 1. Introduction

Wound dressings are of particular interest in wound management. Traditionally used to protect wounds from external contamination, e.g., in the form of gauze [1], there are circumstances, particularly in chronic wounds, where their role is an active one, participating in the wound healing process, either by delivering therapeutic agents or by supporting growth factors that promote re-epithelization [2,3]. It is already accepted that the ideal dressing should be able to remove exudate while maintaining adequate moisture and allowing oxygen to enter the wound [4,5]. Certainly, these features must be combined with other key characteristics, such as biocompatibility, non-toxicity, and non-immunogenicity. They may also be complemented by more specific properties, such as antimicrobial activity or the ability to support vascularization and tissue regeneration [5,6]. The antibacterial activity of wound dressings is an important goal that scientists have extensively tried to achieve through different strategies. For example, silver nanoparticles [7,8] or zinc oxide nanoparticles [9,10,11] are highly appreciated for their antibacterial and antibiofilm activity. Other strategies for improving wound dressings have focused on developing materials that can provide the damaged tissue with adequate moisture, oxygen, and antibiotics. Among the potential candidates in this area, hydrogels have been the subject of much research as wound dressing materials for acute and chronic wounds. From a chemical point of view, hydrogels are polymer networks that have the ability to absorb large amounts of water [12]. The structure of the polymeric network is achieved by a cross-linking step, which can be either covalent (chemical) or physical, and prevents the polymer from dissolving [13]. Due to their wet molecular structure substantiated by their porous framework, hydrogels resemble the physicochemical and biological characteristics of tissues, making them particularly useful in tissue engineering [14]. Moreover, due to their attractive physicochemical and biological properties, biopolymers such as chitosan or alginates, have been extensively studied in the field of controlled drug delivery and wound dressings in various forms, such as hydrogels, sponges, films, or scaffolds [15,16,17]. 

Alginate, usually extracted as sodium alginate, is a polysaccharide composed of (1→4) linked units of D-mannuronic acid (M-block) and L-guluronic acid (G-block) distributed in successive GM blocks [18]. As mentioned above, previous works have centered on nanoparticles embedded in various polymeric networks, including alginate. Although the focus has been on nanoparticles with antibacterial activity [19,20], many studies have also addressed the incorporation of mesoporous silica due to its large surface area and porous framework, which allows it to be loaded with various drug molecules [21,22,23]. This approach would be particularly useful for hydrophilic drugs that tend to burst after administration. For example, vancomycin, whose clinical use dates to 1956 when it was first used to treat penicillin-resistant *S. aureus*, is still one of the most common antibiotics applied for severe infections, also because it has high efficiency against methicillin-resistant *S. aureus* (MRSA) [24]. However, due to its high solubility in aqueous environments, vancomycin has a rather immediate-release behavior, and it has been a challenge to delay its release using mesoporous silica [25]. In addition, vancomycin loaded into stimuli-responsive hydrogels has also been reported to extend the release rate. For example, an oligo (poly-(ethylene glycol)fumarate)/sodium methacrylate (OPF/SMA) hydrogel matrix prolonged the release for up to four days [26], while oxidized hyaluronic acid coupled with adipic acid di-hydrazide (oxi-HA/ADH) reached release equilibrium after three days [27]. In contrast, Sun et al. [25] achieved a much longer and sustained release, up to 30 days, by encapsulating vancomycin-loaded SBA-15 in a chitosan-sodium glycerophosphate-sodium hydrogel (CS-GP-SA). At the opposite pole of this rapid release, vancomycin is characterized by poor tissue penetration [24,28]. Therefore, we hypothesize that incorporating magnetite into an alginate film supplemented with drug-loaded mesoporous silica would improve the rate of vancomycin tissue penetration via a mild hyperthermia effect. As far as we have seen, the field of magneto-responsive alginate-based drug delivery systems is somehow lacking in the field. However, magnetite nanoparticles are already known for their biocompatibility and ability to control drug release and for the possibility of enhancing therapy by hyperthermia. For instance, Richardson et al. [29] demonstrated a significant decrease in living *S. epidermis* upon combining vancomycin with 50 °C hyperthermia. A recently published study [30] also showed that embedding magnetite nanoparticles in chitosan microbeads can stimulate vancomycin release by applying an alternating magnetic field. Similarly, in one of our previous studies, we showed the possibility of enhancing folic acid release upon stimulation using a NdFeB magnet on magnetic alginate film by a magneto-mechanical mechanism of triggering [31]. 

The aim of this research was to obtain a hydrogel-based dressing enriched with vancomycin-loaded MCM-41, whose role is to facilitate a sustained release. In addition, magnetite was used to study the possibility of its homogenous incorporation into the composite film, and to synergistically assist the antimicrobial activity of these wound dressings in infected wounds. This was a first step towards achieving a uniform magnetic film containing mesoporous silica as a drug carrier, to investigate its functionality as a potential wound dressing and to investigate whether the presence of a magnetic field had any influence on the amount of drug released, without studying the hyperthermic effect. Four steps were followed to achieve the aim of the research: (i) synthesis and characterization of a malic acid functionalized magnetite (Fe_3_O_4_/malic), (ii) synthesis and characterization of a mesoporous silica type MCM-41 loaded with vancomycin (MCM-41@Van); (iii) physical mixing of the obtained nanoparticles followed by incorporation in a sodium alginate-glycerol matrix; and, finally, (iv) assessment of the morpho-structural characteristics of the obtained films and testing their functionality in terms of drug release and antimicrobial activity, including the synergistic effect of the components. 

## 2. Results and Discussion

### 2.1. Nanoparticle Characterization

The analytical techniques for each of the obtained nanoparticles have been selected to confirm that specific features required for this study have been achieved. Specifically, magnetite was investigated by X-ray diffraction (XRD) and Fourier transformed infrared spectroscopy (FT-IR). Bare Fe_3_O_4_ has some limitations, as it is prone to oxidation and agglomeration [32], so we chose a one-pot functionalization with a dicarboxylic acid, namely malic acid, which is used in the pharmaceutical and food industries. Although the literature shows several studies on obtaining single-phase magnetite by the coprecipitation method [33], including functionalized-magnetite [34,35], there are not enough data on the functionalization of Fe_3_O_4_ with malic acid. Therefore, we considered X-ray diffraction as an essential analysis to confirm that magnetite is the only crystallographic phase present in our sample, as it shows better biocompatibility than hematite or goethite. In addition, based on the XRD data, it is possible to assess whether the malic acid induces a preferential orientation/growth of the magnetite or whether it simply coats the magnetite without changing the crystalline structure. 

**Figure 1:**
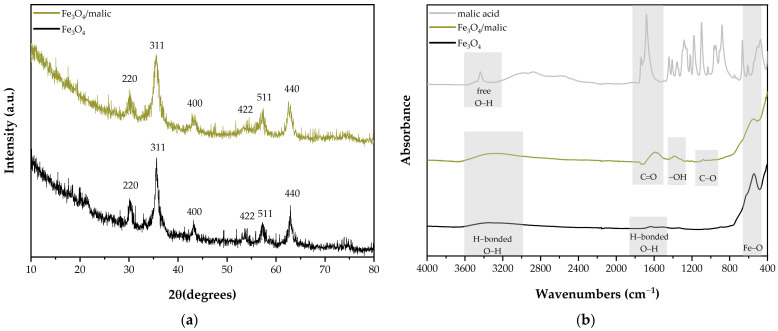
Bare Fe_3_O_4_ and malic-functionalized Fe_3_O_4_ characterization. (**a**) XRD patterns and (**b**) FT-IR spectra of the magnetite samples compared to pure malic acid.

Figure 1a shows the stacked XRD patterns of a bare Fe_3_O_4_ and malic-functionalized Fe_3_O_4_ (Fe_3_O_4_/malic). Both diffractograms are consistent with previous studies, where magnetite was obtained by coprecipitation and showed strong diffraction peaks in the 10–80° 2θ range. According to COD 96-900-5842, the peaks correspond to the (220), (311), (400), (422), (511) and (440) diffraction planes, characteristic of cubic Fe_3_O_4_. Moreover, the sharp appearance of the peaks, indicates that the considered samples have a high degree of crystallinity, which is maintained after functionalization. Compared to pristine Fe_3_O_4_, Fe_3_O_4_/malic did not show any additional peak, magnetite being the only phase present. This result is in line with a previous study, where malic acid was used to coat Fe_3_O_4_ nanoparticles [36]. However, a slight peak broadening can be observed, probably due to a slight decrease in crystallite size, which was also observed by Alzoubi et al. [37] in a recent study where Fe_3_O_4_ was coated with citric acid.

Infrared spectra of malic acid, Fe_3_O_4_ and Fe_3_O_4_/malic (Figure 1b) were recorded to confirm whether Fe_3_O_4_ has been successfully coated. The surface functionalization was an important target, as it prevents the agglomeration of the nanoparticles, thus allowing a homogeneous particle dispersion within our final system, the magnetic film. Fe_3_O_4_ and Fe_3_O_4_/malic samples revealed a mutual peak corresponding to Fe–O stretching vibration, at ~550 cm^−1^, normally reported in previous studies [38,39]. The large absorption bands centered around 3345 cm^−1^, present in both samples, are assigned to –OH stretching vibrations, while Fe_3_O_4_ additionally presents a very faint absorption band at 1630 cm^−1^, attributed to –OH bending vibrations. Fe_3_O_4_/malic, on the other hand, shows a new slight intense absorption peak at 1594 cm^−1^, a slightly less intense band at 1389 cm^−1^ and an exceedingly small signal at 1081 cm^−1^. Compared to the spectrum of malic acid, which showed a strong peak specific for carbonyl stretching vibrations (1738 cm^−1^), the highest peak of the Fe_3_O_4_/malic sample (1594 cm^−1^) indicates a shift of the carbonyl group to a much lower frequency, due to interactions between Fe_3_O_4_ and malic acid probably via carboxylate ions [40]. The same behavior was exhibited in a previous study after coating Fe_3_O_4_ with succinic acid and citric acid [41], suggesting that rigid structures of the coating agent may result in smaller shifts than less rigid coating agents. Carbonyl band broadening is also a good indicator of the involvement of carbonyl groups in hydrogen bonding. In addition, the signals that have appeared at 1389 and 1081 cm^−1^ correspond to symmetrical stretching vibrations of the carboxyl and hydroxyl groups of malic acid.

Regarding mesoporous silica and its proposal as a nanocarrier-diffusional barrier of the Van from the alginate gel, the critical investigations included the confirmation of drug loading in the material, as well as both unloaded and loaded-MCM-41 textural characteristics, especially the surface area and pore volume. Therefore, FTIR and Raman spectra were examined and corroborated with N_2_ adsorption-desorption isotherms of bare MCM-41 and vancomycin-loaded MCM-41 (MCM-41@Van).

**Figure 2:**
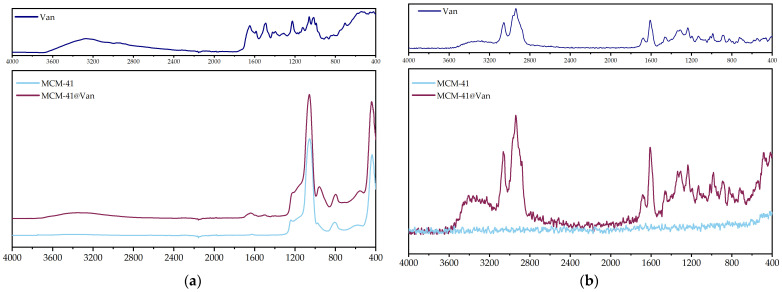
Comparative spectra of pure vancomycin hydrochloride (Van), MCM-41 and MCM-41@Van: (**a**) infrared absorbance vs. wavenumbers spectra, (**b**) intensity vs. Raman shift spectra.

FT-IR spectra were obtained to demonstrate vancomycin loading in the MCM-41 type silica (Figure 2a). Pure vancomycin hydrochloride (Van) showed the broad, strong absorption band of the hydroxyl groups at 3270 cm^−1^ and the carbonyl stretching vibration at 1648 cm^−1^ as a strong peak, typical for lactam rings. For MCM-41, the FT-IR spectrum revealed specific absorption bands at 815 and 1077 cm^−1^, typical of silanol and siloxane functional groups. In the case of MCM-41@Van, there were no significant differences except for the peak at 1648 cm^−1^ of the lactam ring present in vancomycin. Other bands corresponding to vancomycin could not be confirmed due to high Si–O and Si–O–Si absorption bands overlapping those associated with vancomycin. However, Raman spectroscopy (Figure 2b) was further employed as a complementary technique, the intensity of the Si-O–Si vibrations between 1010–1095 cm^−1^ being significantly lower than in infrared, which allowed us to identify the fingerprint region of vancomycin. Finally, it was confirmed that MCM-41@Van had been successfully loaded.

**Figure 3:**
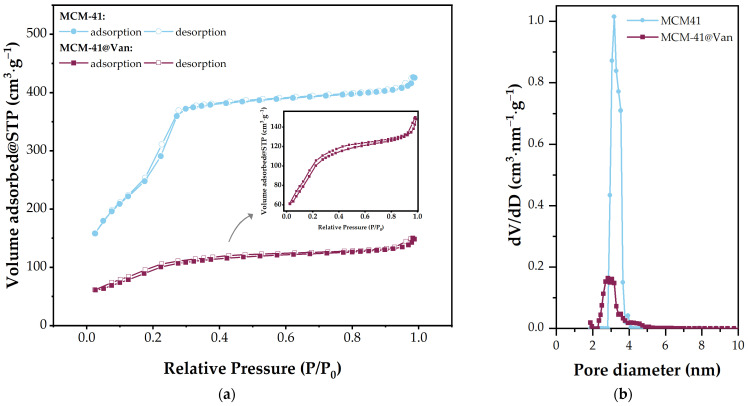
(**a**) Nitrogen adsorption/desorption isotherms and (**b**) pore size distribution of MCM-41 and MCM-41@Van.

The nitrogen adsorption-desorption isotherms and textural characteristics of MCM-41 and MCM-41@Van are depicted in Figure 3 and Table 1, respectively. MCM-41 exhibits a conventional type IV isotherm without hysteresis. The inflection that occurs at less than 0.3 p/p_0_ value indicates a capillary condensation process occurring in the material. Compared to the unloaded sample, the inflection point of MCM-41@Van does not change as steeply as in the case of unloaded MCM-41. As can be seen in the inset of Figure 3a, the shape of the isotherm also remains the same, indicating that the drug loading process did not affect the pore structure. The synthesized MCM-41 had a large surface area (1233 m^2^/g), which was significantly reduced upon vancomycin loading, decreasing 3.49-fold. Similarly, the pore volume reduced 2.97-fold. Furthermore, the drug loading amount was calculated as previously reported by [42], using the observed pore volume reduction and considering a vancomycin molar volume of 875 cm^3^·mol^−1^ (determined on the basis of a molecular weight of 1485.71 g·mol^−1^ and a density of 1.7 g·cm^−3^ for vancomycin). Assuming that the pore reduction of 0.4234 cm^3^·g^−1^ is ascribed to the volume filled by vancomycin molecules, this would correspond to 0.719 g of vancomycin per gram of MCM-41@Van, giving a loading percentage of approximately 71.9%, which is much higher than the theoretical loading of 30%. This phenomenon could appear due to capillary condensation of vancomycin on the surface of MCM-41, sealing off areas of the pores from further loading, but also for nitrogen adsorption during BET analysis. The same effect was observed by Katsiotis et al. for celecoxib [42]. In terms of average pore diameter, MCM-41@Van exhibits a 1.12-fold lower value. By comparing the pore size distributions from Figure 3b, it is easy to see that a shift towards smaller pore sizes occurs for the loaded-MCM-41. The decrease in peak heights is also correlated with the decrease in the volume of the pores.

**Figure 4:**
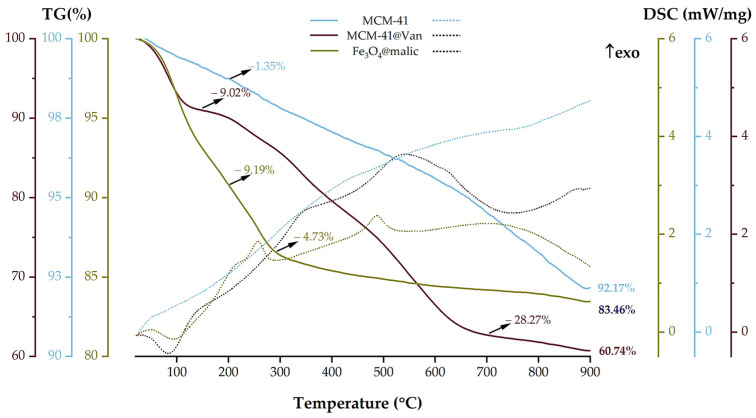
Thermal analysis of the nanoparticles. Solid line TGA, dot line DSC.

Figure 4 gives the thermal analysis between 23 and 900 °C using thermogravimetry (TGA) and differential scanning calorimetry (DSC). 

Fe_3_O_4_/malic loses 9.19% of its initial mass up to 200 °C, which is specific to the elimination of adsorbed water molecules. The process is accompanied by an endothermic effect with a minimum at 93.5 °C. At higher temperatures, the degradation of the organic molecules takes place in a series of decomposition and oxidation reactions. The sample loses a further 4.73% of its mass at 320 °C, with a pronounced exothermic effect at 257 °C, as shown by the DSC curve. Most likely, an oxidation process of malic acid occurs. After 320 °C, the sample slowly loses another 2.62% of its mass, which is due to the combustion of carbonaceous mass and the elimination of the –OH surface moieties. The strong exothermic effect at 486.9 °C is specific for the transformation of maghemite to hematite [43,44]. The residual mass at 900 °C is 83.46%. 

In the case of the MCM-41, two temperature ranges are given in the literature: up to 200 °C for the elimination of adsorbed water molecules and between 200 °C and 900 °C for the condensation of silanol groups. The TGA analysis of bare MCM-41 showed these two losses of mass, the first between room temperature and 200 °C, when the sample loses 1.35% of its initial mass, representing the elimination of physically adsorbed water molecules from both the outer surface and the inner surface of the pores. More specifically, up to 100 °C, the sample loses the water that filled the pores with little or no surface interactions (0.65%). In the temperature interval between 100 °C and 200 °C, MCM-41 showed an additional weight loss of 0.70%. This loss is attributed to water molecules bound to the surface of MCM-41, which required additional energy to break free of the water. The release of these two types of water molecules is indicated by the endothermic effects present on the DSC curve, with a minimum at 55.1 °C and another at 119.0 °C [45]. Finally, in the temperature range 200–900 °C, the water is eliminated by condensation of the silanol moieties, the process being responsible for silica formation and densification. The process was slow and of low intensity, giving the continuous mass loss aspect of the TG curve in this interval (6.57%) [46]. The residual mass recorded at 900 °C was 92.17%. In contrast, the loaded mesoporous silica showed several mass losses due to the presence of the antibiotic. Up to 150 °C, MCM-41@Van loses 9.02% of its initial mass, with the elimination of water residues absorbed on the particle surface during the loading process. Immediately after 150 °C, vancomycin begins to degrade in an oxidative-degradative process, with the sample losing a further 28.27% of its mass. While water elimination takes place in an endothermic process with an endothermic peak at 84.0 °C, the DSC curve associated with vancomycin degradation shows an overlap of several exothermic reactions with two distinct peaks present at 352.0 °C and 540.3 °C. The residual mass at 900 °C is 60.74%. This value allowed the quantification of the vancomycin present in our sample, which was 34.10%, closely to our calculated amount. The main data obtained from the TGA-DSC analysis are also summarized in Table 2. 

**Figure 5:**
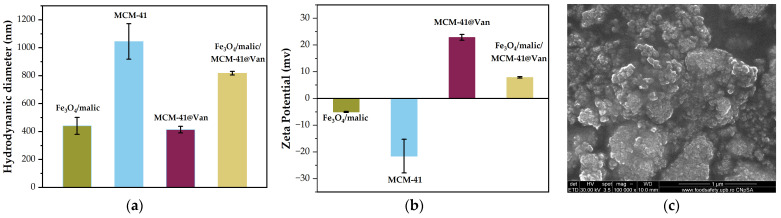
(**a**) Hydrodynamic diameter, (**b**) zeta potential for Fe_3_O_4_/malic, MCM-41 and MCM-41@Van measured by dynamic light scattering technique and (**c**) Fe_3_O_4_/malic acid SEM image (100,000× magnification).

Finally, the hydrodynamic diameter and zeta potential were measured for the magnetite, unloaded, and loaded mesoporous silica, as well as for the mixture obtained after physically grinding the two types of nanoparticles (Figure 5a,b). Fe_3_O_4_/malic showed an average hydrodynamic diameter of 441 nm. Considering the low zeta potential (−5.02 mV) of the Fe_3_O_4_/malic, the repulsive forces are low, and the magnetic attraction cannot be compensated and, thus, a strong agglomeration occurs, which gives a hydrodynamic diameter over 400 nm even if these nanoparticles are much smaller, as can be seen in Figure 5c. In the case of the mesoporous material, the results demonstrate that the addition of vancomycin significantly decreases the hydrodynamic diameter, from 1045 nm to 414 nm, indicating a reduction in the interaction between the vancomycin-loaded MCM-41 support and the solvent (ethanol). These observations are correlated with the polydispersity index (PDI) values, which are not affected by the presence of the bioactive substance. However, since both PDI values are greater than 0.4, the size distributions of the pristine and vancomycin-loaded MCM-41 supports are polydisperse. Furthermore, the loading of vancomycin is demonstrated by the zeta potential values, which vary from approximately −22 mV to +23.7 mV due to the positively charged vancomycin molecules. The negative zeta potential of Fe_3_O_4_/malic (−5.02 mV), on the other hand, indicates a negative surface, leading to its physical association with the positively charged surface of vancomycin-loaded MCM-41. The mix of the nanoparticles showed a positive zeta potential (7.87 mV) which was expected due to the high positive zeta potential of the loaded MCM-41. This is of great help in combining the nanoparticles with the alginate, which is an anionic polymer.

### 2.2. Characterization of the Vancomycin-Loaded Magnetic Alginate Film

The films obtained after oven drying were visually inspected. The simple alginate/glycerol film, labeled AG, was transparent. While the film with embedded vancomycin-loaded mesoporous silica (AGSi) retained its transparent aspect with a slight milky texture, the magnetic film, to which both the MCM-41@Van and Fe_3_O_4_/malic mixture were added (AGSiM), was dark brown. Both films with embedded nanoparticles showed very good macroscopic homogeneity, which was maintained after cross-linking with CaCl_2_. In addition, the films were smooth and flexible after cross-linking. Further, the samples were evaluated for their structural and morphological homogeneity at microscopic level.

**Figure 6:**
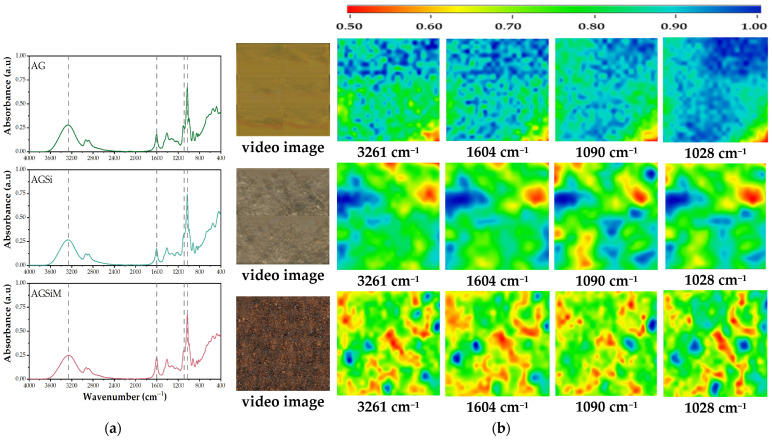
(**a**) FT-IR spectra of cross-linked films. Dashed lines indicate the selected wavenumbers for FT-IR mapping investigation (3261 cm^−1^, 1604 cm^−1^ 1090 cm^−1^ and 1028 cm^−1^; (**b**) FT-IR microscopy at selected wavelengths. Red areas indicate the highest absorbance, while blue areas correspond to the lowest absorbance.

The use of infrared spectroscopy is effective in determining the structural homogeneity of the samples. As can be seen from Figure 6a, the spectra are quite similar in terms of intensity and absorption bands. Therefore, FTIR microscopy was used as a complementary technique to check for possible heterogeneities in the sample due to the embedding of the nanoparticles. Characteristic bands of alginate can be seen in all three films. The absorption band centered at 3261 cm^−1^ is specific to hydroxyl stretching vibrations and is closely followed by less intense peaks at 2943 and 2888 cm^−1^ corresponding to saturated carbon-hydrogen bonds. The intense band at 1028 cm^−1^ is related to C-O-C stretching vibrations, some studies referring to it as the glycosidic bond from the alginate molecule, while the shoulder at approximately 1100 cm^−1^ corresponds to α-glycosidic bond. Starting from the infrared spectra, the absorption bands at 3261 cm^−1^, 1604 cm^−1^, 1090 cm^−1^ and 1028 cm^−1^ were selected as a reference to study the structural homogeneity of the films. Images from Figure 6b show the infrared mapping performed for the corresponding wavelengths. In the case of the AG sample, a very good homogeneity is observed, with similar peak intensities over the entire investigated surface. In the case of the vancomycin-loaded silica sample embedded into the alginate film (AGSi), some areas with more intense absorptions are observed but the overall FTIR maps recorded at the four wavelengths are almost superimposed, which means a good homogeneity at the micrometric scale. In the case of the alginate film containing both MCM-41@Van and Fe_3_O_4_/malic, the areas of lower intensity (blue areas) correspond to particle agglomerates, as could be distinguished from the video image of the area under examination. Overall, the films can be considered structurally homogenous, and the embedding of the nanoparticles did not alter the structure of the alginate matrix.

**Figure 7:**
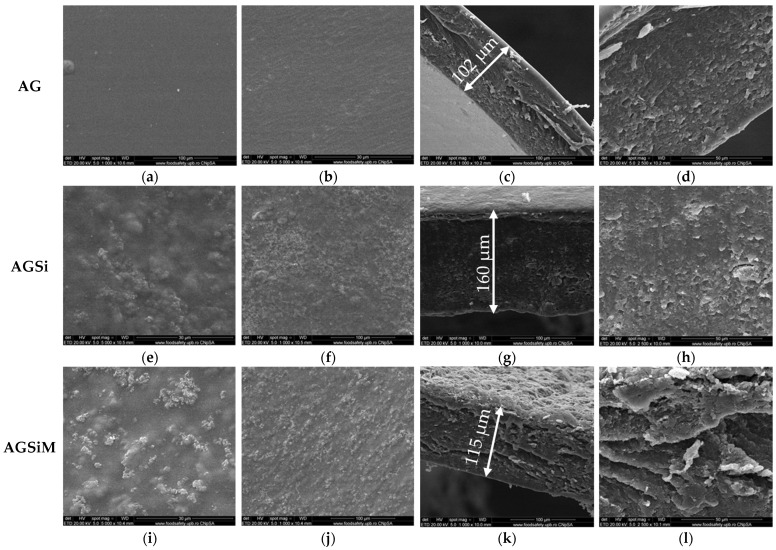
SEM images of AG (**a**–**d**) AG, AGSi (**e**–**h**) and AGSiM (**i**–**l**) films. Images (**a**,**e**,**i**) represent surface of the films at 1000× magnification; images (**b**,**f**,**j**) represent surface at 5000× magnification; images (**c**,**g**,**k**) are cross-sections of the films at 1000× magnification, while images (**d**,**h**,**l**) are cross-sections at 2500× magnification.

SEM micrographs (Figure 7) revealed a distinct surface view between the simple alginate film and films with embedded nanoparticles. Compared to the AG film, which has a predominantly smooth surface, AGSi and AGSiM films exhibit solid particles on the surface. Both types of nanoparticles presented good compatibility with the alginate matrix. This was expected since prior DLS measurements showed the positive zeta potential of the nanoparticles, which could help in creating strong ionic interactions with the alginate. Hydroxyl groups present on both types of nanoparticles, derived either from malic acid in the case of Fe_3_O_4_/malic, or from vancomycin in the case of MCM-41@Van could also lead to the formation of hydrogen bonds with the hydroxyl groups present in the alginate. Cross-section analysis of the films allowed the surfaces to be viewed from a different angle, where it can be seen that the AGSi film has a more compact topography, whereas AGSiM has a rougher surface, giving a more aerated and porous appearance. In addition, the cross-section view of AGSiM shows ravines, which are probably the result of the cutting of the films. The AGSi film is approximately 160 µm thick compared to AGSiM film, which is only 115 µm. 

**Figure 8:**
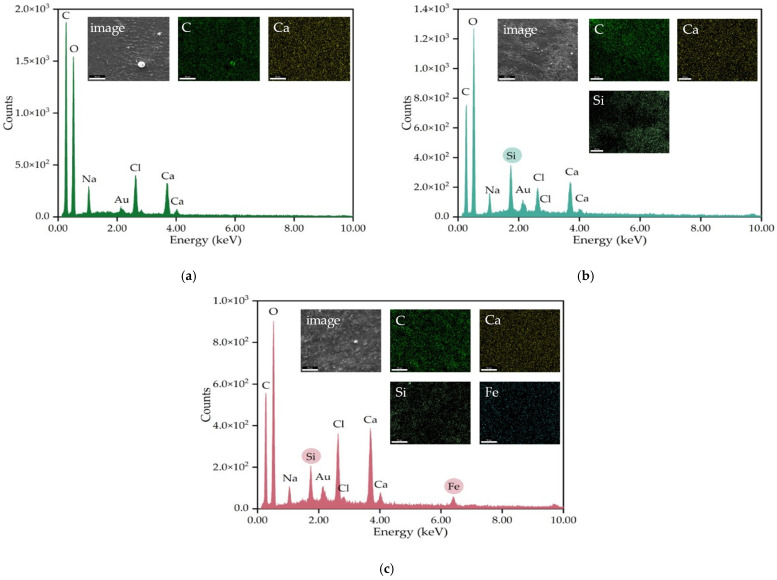
Energy dispersive X-ray analysis (EDX) and elemental mapping of AG (**a**), AGSi (**b**) and AGSiM (**c**) films.

EDX analysis and elemental mapping were carried out to complement SEM analysis and to verify the homogeneity of the nanoparticles in the sample. As can be seen from the EDAX spectra (Figure 8), all the samples have a high carbon and oxygen content due to the alginate. The amount of oxygen increases with the incorporation of nanoparticles, which is normal, as these are metal oxide nanoparticles. Sodium, chlorine, and calcium are present in all three films due to cross-linking with calcium chloride, and gold due to sample preparation for analysis. The mapping of the elements showed a very good uniformity for calcium, which has the role of forming the cross-linked polymeric structure by replacing sodium ions in sodium alginate. AGSi has a peak corresponding to silicon in its EDX spectrum, while elemental mapping indicates a reasonable dispersion of silicon in the polymeric mass, silicon being complementary to carbon. In the case of AGSiM film, the silicon distribution is much better than the AGSi film, while the iron is also well dispersed in the film. In fact, the two microscopy tools (FTIR and SEM) reveal a very good homogeneity, offering the premise of compositional and functional reproducibility.

**Figure 9:**
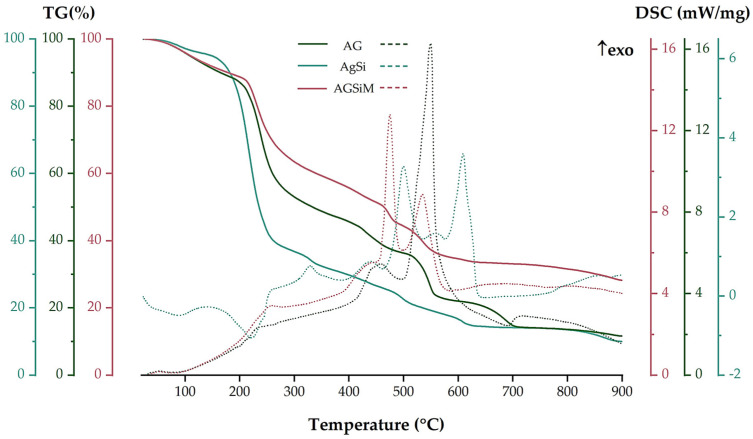
TGA-DSC of AG, AGSi and AGSiM films.

Figure 9 depicts the TGA-DSC plots for the three films. TGA curves revealed a four-stage mass reduction for each of the films. All films show good stability at temperatures between 37–45 °C, temperatures associated with the therapeutic effect of hyperthermia. Further, the films exhibited a mass loss of up to 200 °C, which is related to the loss of water molecules physically bound to the film structure. In all cases, the DSC curve showed endothermic effects occurring at 81.26, 87.80 and 79.5 °C for AG, AGSi and AGSiM, respectively. In the case of the AGSi film, the loss of mass corresponding to the break of water molecules occurs at slightly lower temperatures, at around 130 °C, compared to 200 °C for AG and AGSiM. This is most probably due to the presence of vancomycin in the film bulk, which also starts to decompose up to 200 °C. Furthermore, it is also consistent with the TGA-DSC analysis of vancomycin-loaded silica, where the material lost water up to 150 °C, after which vancomycin degradation began. Even if the AGSiM film also presents vancomycin, the magnetite presumably acts as a shield for the antibiotic. 

With increasing temperature, all films continued to lose mass. AG revealed a short temperature range between 200–300 °C, where it decomposes thoroughly, losing 32.92%, this being an exothermic process with a peak at 235 °C, which indicates the presence of degradation and oxidation processes of the organic part of the sample. The same pattern is observed for the AGSiM sample, which loses 24.05% of its mass in the same temperature range; the process is also accompanied by an exothermic effect peaking at 258.8 °C. On the other hand, the AGSi sample loses another 58.03% of its mass, with an endothermic peak at 221.4 °C. This is due to the fragmentation of the organic phases present in the sample. 

Most of the changes in all three films occur between 300–700 °C, with several exothermic effects. In the case of the simple alginate film, two mass losses of 17.88% and 14.30% occurred, accompanied by two strong exothermic effects with maxima at 457.4 °C and 549.4 °C, respectively. The peak at 549.4 °C corresponds to the burning of the carbonaceous mass. In the case of the AGSi film, oxidation processes dominate after 300 °C, as indicated by several exothermic effects occurring at 328.8, 439.4, 499.7 and 609.0 °C. Nevertheless, the mass loss is uniform, with an overall reduction of 23.61%. The AGSiM film loses another 30.06% of its mass with three maximum peaks at 437.0, 474.8 and 534.4 °C. Finally, at 900 °C, the residual masses of AG, AGSi and AGSiM are 11.64%, 9.97% and 28.21%, respectively. 

### 2.3. Performance of Vancomycin-Loaded Magnetic Alginate Film

#### 2.3.1. Wound Fluid Uptake and Water Vapor Transfer Rate

**Figure 10:**
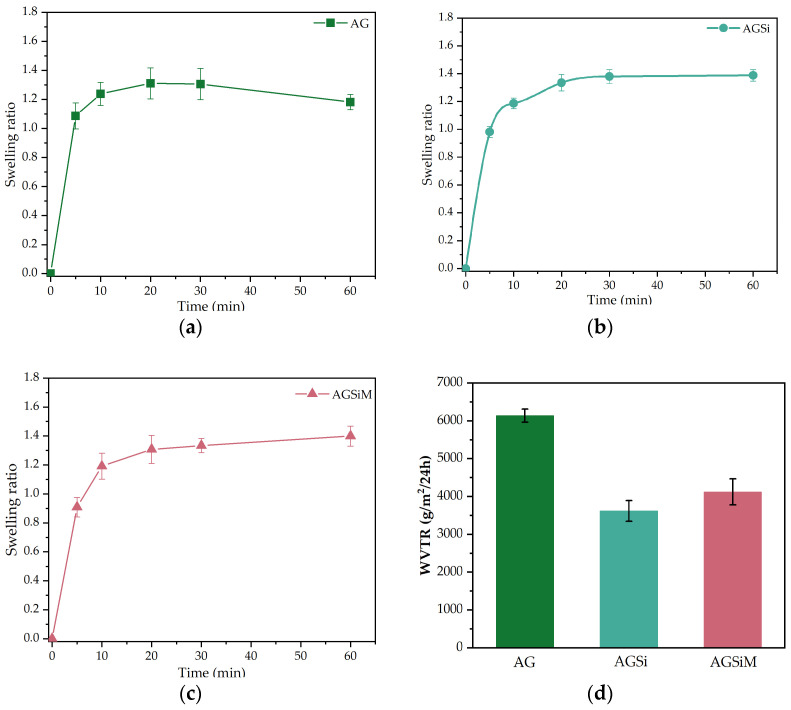
Wound fluid uptake for (**a**) AG, (**b**) AGSi and (**c**) AGSiM. Water vapor transmission rate for the three films (**d**). All results are expressed as mean value ± standard deviation, *n* = 3.

The ability to absorb exudate was tested by examining the swelling behavior of the films after immersion in a simulated wound fluid (SWF) prepared according to a procedure previously described in reference [47]. The swelling behavior was expressed as the swelling ratio, and the results obtained for each film are plotted separately in Figure 10a–c.

The swelling ratio of all the films rapidly increased within the first 10 min, indicating a rapid wound fluid uptake. However, the AG film reached the maximum value after approximately 30 min, while films containing nanoparticles continued to slightly absorb water up to one hour. As for the AG film, after one hour, there is a slight decrease in mass, which can be attributed to the observed degradation that occurred while taking the measurement. Small fragments of the film split up, inducing a decrease of the weighed mass. Although we initially thought this was an error due to the use of very sharp tweezers to remove the films from the liquid, the same behavior was propagated in all three replicates of the experiment, which indicates a lower resistance of the film compared to the films obtained by incorporating nanoparticles. Further weightings were assessed after 24 h and 48 h to investigate the stability of the films. After completing the swelling, the recorded masses were 53.6 ± 2.39 mg, 76.7 ± 5.1 mg and 56.8 ± 2.2 mg for AG, AGSi and AGSiM films. After 24 h, the recorded masses were 49.3 ± 2.0 mg (AG), 76.0 ± 1.3 mg (AGSi) and 50.4 ± 3.0 mg (AGSiM), respectively 39.4 ± 1.8 mg (AG), 68.2 ± 3.6 mg (AGSi) and 47.7 ± 2.9 mg (AGSiM) after 48 h. The highest mass difference (14.1 mg) occurs for the AG sample, indicating a degradation of the film at higher rate than the AGSi and AGSiM samples. AGSi sample exhibited an 8.5 mg mass loss, while the AGSiM sample, on the other hand, loses 9.1 mg of its mass in 48 h. It can see that the mass of the samples after 48 h is lower than that at 24 h, most probably because some of the drug is released, but also because of the dissolution of the alginate film. In conclusion, the films containing nanoparticles are more stable than the alginate films in contact with the simulated wound fluid.

The water vapor permeability of hydrogels prevents the accumulation of fluid in an exuding wound, so the WVTR is a good indicator of this important property, which has a major impact on the wound healing process [48]. Normal skin has a WVTR of 204 g/m^2^/day. However, in wounded skin, this rate can increase to 279 g/m^2^/day, and in excessive wounds, it can increase up to 5138 g/m^2^/day [49,50]. The WVTR values for AG, AGSi and AGSiM samples were 6139 ± 172 g/m^2^/day, 3619 ± 275 g/m^2^/day, and 4122 ± 345 g/m^2^/day, respectively. The addition of nanoparticles reduces the WVTR of the films, most likely because their presence generates a more compact structure and, thus, a more complicated path for water molecules to pass through the film, and therefore limits the amount of water that can permeate compared to the bare film. Although the slightly lower WVTR value of the AGSi film could be explained by the more compact structure, as shown in the SEM images, there is no significant difference between the means when the one-way ANOVA test is applied to the AGSi and AGSiM groups. Dividing the WVRT by the number of hours in a day, the water vapor transmission would be approximately 151 g/m^2^/h and 172 g/m^2^/h, respectively, which translates into the ability to transfer 15.1 mg of water per cm^2^ and 17.2 mg of water per cm^2^ for AGSi and AGSiM, respectively. This is an important feature since, according to the wound fluid uptake study, the AGSi and AGSiM films absorbed 44.8 mg and 27.9 mg SWF in one hour for the same area of the films, suggesting that a significant proportion of the absorbed exudate can be removed from the wound site thanks to its permeability and thus compensating for the quite low swelling capacity of these films. 

#### 2.3.2. Vancomycin Release Behavior

**Figure 11:**
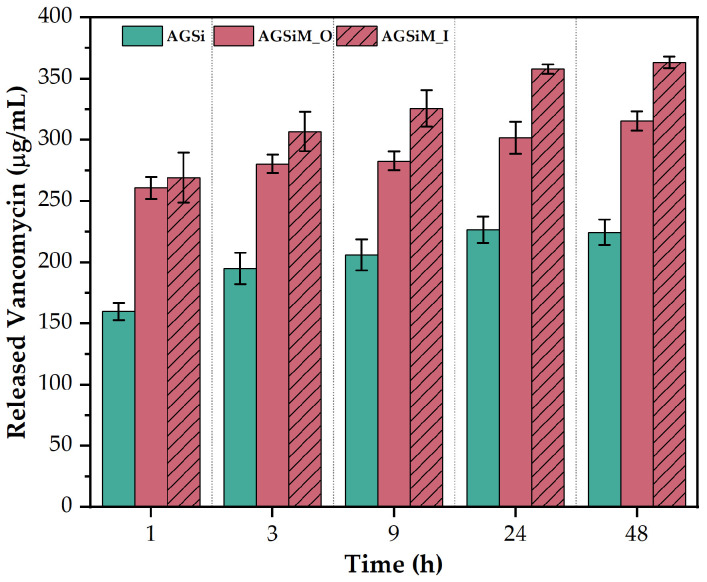
Concentration of vancomycin released from AGSi film, unstimulated AGSiM film (AGSiM_O) and stimulated AGSiM film (AGSiM_I). Data is represented as mean ± standard deviation from three independent experiments.

As shown in Figure 11, all films release vancomycin over 48 h, with significant differences between the AGSi film and the magnetite films. In the first hour, the release medium shows 160 ug/mL, corresponding to 1.6 mg of vancomycin released, and after 24 h reaches approximately 227 ug/mL, corresponding to 2.27 mg of vancomycin released from the film. The unstimulated AGSiM film releases 1.63 times and 1.33 times more vancomycin at 1 h and 24 h, respectively, compared to AGSi. While stimulation of release by the magnetic field did not significantly improve vancomycin release in the first three hours, the difference between the means at 9, 24 and 48 h is significant at the 0.05 level using the Tukey test. After 48 h, the average vancomycin concentration is 224 ug/mL for AGSi, 315 ug/mL for unstimulated AGSiM and 363 ug/mL for magnetically stimulated AGSiM. As the AGSi sample releases 2.24 mg total amount of vancomycin and the unstimulated AGSiM sample releases 3.15 mg total amount of vancomycin, this also correlates with the stability test conducted over 48 h, where a slightly lower mass loss was observed for the AGSi sample. A similar pattern for vancomycin release was observed by Mohapatra et al. from chitosan microbeads embedded with magnetic nanoparticles [30].

#### 2.3.3. Antimicrobial Activity

##### Qualitative Evaluation of the Antimicrobial Activity

Antimicrobial activity was qualitatively evaluated by measuring the diameters of the inhibition zones around the samples. The data results are presented in Table 3 as mean values ± SD (standard deviation). 

The qualitative results show that all alginate-based films have exhibited antimicrobial activity against the tested strains. The Gram-positive bacteria were more sensitive than *C. albicans*, and the films enhanced with Van-loaded MCM-41 (AGSi and AGSiM) showed the most significant antimicrobial activity. 

Another study [51] reported similar results in the antibacterial activity of a vancomycin-loaded alginate coating against *S. aureus* (35 mm) and demonstrated the role of alginate in decreasing vancomycin release. Kurczewska et al. [52] also demonstrated the antibacterial activity of halloysite nanotubes against *Staphylococcus* sp., *Streptococcus* sp. and *E. faecalis* ATCC 29212. After 24 h, 70% of the vancomycin was released from the silica materials and 44% from the alginate. Otherwise, the alginate prolonged the antibiotic release, and only the immobilized vancomycin in alginate showed significant antibacterial activity. 

The alginate films enriched with MCM-41@Van and magnetic nanoparticles functionalized with malic acid had better antimicrobial activity, especially against *E. faecalis* and *S. aureus*, confirming the synergic effect between bioactive compounds [53,54,55]. 

##### Quantitative Evaluation of the Anti-Adherence Capacity of the Alginate-Based Films

**Figure 12:**
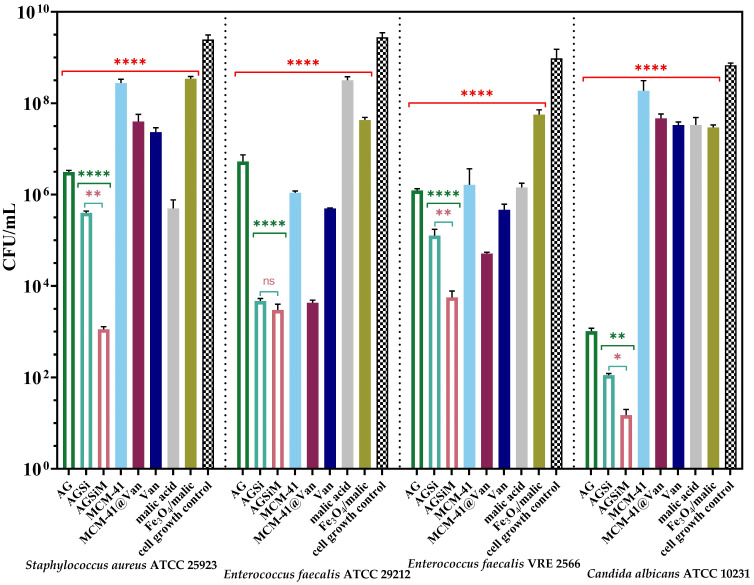
Graphical representation of CFU/mL values of tested strains to evaluate the release of bioactive compounds from alginate-based films into the broth media after 24 h. The differences between groups were compared using one-way ANOVA and Holm-Šídák’s multiple comparison tests and are considered statistically significant (ns—not significant, * *p* < 0.05; ** *p* < 0.005; **** *p* < 0.0001).

**Figure 13:**
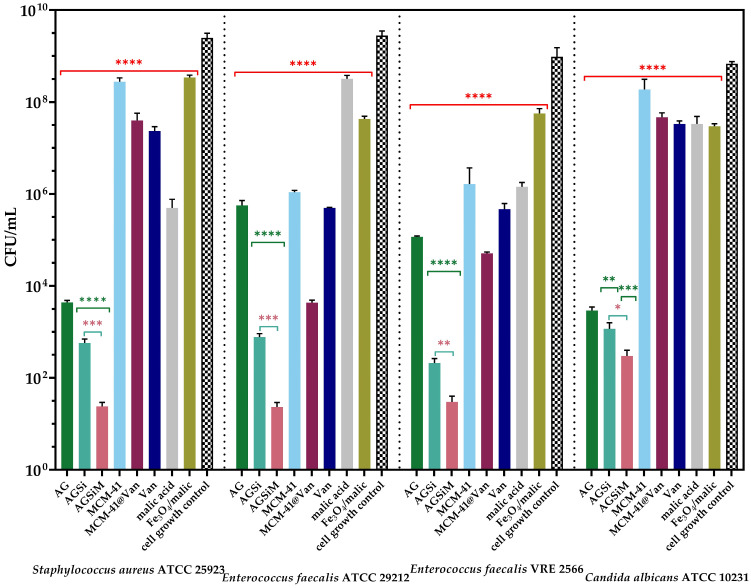
The anti-adherence capacity of alginate-based films against the tested strains. The data results were compared using one-way ANOVA and Holm-Šídák’s multiple comparison tests (* *p* < 0.04; ** *p* < 0.004; *** *p* < 0.006; **** *p* < 0.0001).

Skin/tissue infections are defined as damage caused by pathogens, with variable resistance and severity [56]. The most characteristic feature of wound infections is their ability to form a biofilm, which affects the healing process by reducing the efficacy of antibiotics and inducing resistance of the microorganisms to treatment [57]. Moreover, the wound-healing process is disrupted and delayed [58]. The anti-biofilm capacity of the alginate-based films studied is presented in Figure 12 and Figure 13.

The quantitative assays confirmed the results in Figure 12, showing the release of the biologically active compounds from the samples into the media broth. The films showed a decrease of at least three logarithmic units, suggesting the ability of the samples to inhibit cell proliferation in the liquid media. The hydrogel films enriched with bioactive compounds presented a significant antimicrobial activity, which implies the achievement of a synergic effect between alginate and MCM-41@Van, respectively, and alginate, MCM-41@Van and Fe_3_O_4_/malic acid. 

Moreover, according to nanoparticle characterization (Figure 2 and Figure 3), MCM-41@Van presented vancomycin both in its pores and on its surface. This is an advantage in terms of antimicrobial activity because the encapsulated drug is released slower than the unencapsulated drug. Thus, after a burst release, most probably caused by the available drug on the surface, the drug from the pores is also released and maintains a proper concentration of Van. Therefore, these systems have better antimicrobial activity [45,53,59,60]. Otherwise, the bioactive compounds from AGSi and AGSiM films showed the highest sensitivity against *C. albicans*. AG films also presented higher antimicrobial activity against yeast than other strains or compounds tested. 

An inhibitory effect was observed against vancomycin-resistant bacteria. The alginate-based films significantly affected the development of *E. faecalis* VRE 2566, reducing cell growth by more than three CFU/mL units. Furthermore, the growth of standard strains was reduced by more than five CFU/mL units. The strongest synergism was observed for *C. albicans* and *S. aureus* when the inhibition was much higher in the case of AGSiM compared with AGSi, as well as with any bioactive compound.

Figure 13 shows a significant decrease in the CFU/mL values of the alginate-based films, suggesting an ability to inhibit the adherence of the bacteria to their surfaces. The alginate films presented a moderate bacteriostatic effect against *E. faecalis* sp., but the enriched films showed the highest sensitivity against this strain. The AGSi and AGSiM films showed reduced viable colony-forming with more than six units. Furthermore, the incorporation of Fe_3_O_4_/malic acid significantly improves the antimicrobial activity of the films. Malic acid has antibacterial activity against Gram-positive and Gram-negative bacteria [61] and. when combined with vancomycin, a synergic effect was obtained that determined the highest sensitivity of *Pseudomonas aeruginosa* [62]. Furthermore, the use of malic acid as a passivating agent by grafting it onto the Fe_3_O_4_ surface makes the resulting magnetic nanocarriers a more biocompatible, water dispersible and payload for drugs [36]. 

Based on the anti-adherence capacity and release results, it can be assumed that alginate slows the release of bioactive compounds, especially vancomycin. Similarly, enriched alginate-based films present non-adherent surfaces and can potentially be used as good candidates for wound dressings. The best anti-adherent capacity was obtained for the hybrid AGSiM films, which is 5 to 6 units higher than the pure Van, and 8 units compared with the cell growth control.

## 3. Conclusions

The aim of the present work was to obtain alginate-based films enhanced with MCM-41 and Fe_3_O_4_ to improve the release profile of vancomycin from an immediate-release type to a potentially sustained and controlled release. The first step consisted in the co-precipitation synthesis of a malic acid functionalized magnetite as a stimuli-responsive component to improve tissue penetration of vancomycin. XRD analysis confirmed a monophasic material, with magnetite being the only phase present, while FT-IR analysis confirmed the surface functionalization. The second step consisted of the sol-gel synthesis of MCM-41, followed by a vancomycin loading step using a vacuum-assisted method. BET analysis confirmed a material with high specific surface area and porosity, that were further significantly decreased after vancomycin loading. The successful loading of the antibiotic into MCM-41 was confirmed by FT-IR and FT-Raman analysis. Finally, the hydrogel-based films with embedded nanoparticles were obtained. As shown by FT-IR and SEM microscopy, the nanoparticles showed good dispersion throughout the film and were highly homogeneous both structurally and morphologically. The performance of the films was assessed by investigating wound fluid uptake and water vapor transmission rate, which are important properties for wound dressings. Simulated wound fluid rapidly penetrated the films, and the films were swollen within one hour. The water vapor transmission rate correlated well with the swelling capacity, suggesting good wound fluid transfer, which is essential for a normal wound healing process. 

The obtained films present good premises for their use as wound dressings both from a morpho-structural point of view and antimicrobial activity enhanced by the synergy between the constituent components. The more complex system containing Fe_3_O_4_/malic and MCM-41@Van embedded into the alginate film highlights a strong synergism and, thus, the strongest antimicrobial and antibiofilm activity. The AGSiM film exposed to a magnetic field generated by a NdFeB magnet releases 1.63 times and 1.33 times more vancomycin compared to AGSi. However, the study should be taken further by evaluating the role played by the application of alternating magnetic fields at different biomedically acceptable frequencies and by monitoring the ability to generate heat, inducing the hyperthermic effect.

## 4. Materials and Methods

### 4.1. Materials

Iron (III) chloride, iron (II) sulphate, sodium alginate, calcium chloride, cetyltrimethylammonium bromide and vancomycin hydrochloride from *Streptomyces orientalis* were purchased from Sigma Aldrich. Ethanol, tetraethyl orthosilicate and glycerol were purchased from Merck, while ethanol was acquired from Carl Roth and sodium hydroxide from Honeywell. 

The antimicrobial activity was performed using Nutrient Broth No. 2 (NB), Mueller-Hinton Broth (M-H), Sabouraud Glucose Agar with Chloramphenicol and agar purchased from Sigma-Aldrich (St. Louis, MI, USA). All strains tested in this study were provided by the Microorganisms Collection of the Department of Microbiology, Faculty of Biology & Research Institute of the University of Bucharest (Bucharest, Romania).

### 4.2. Synthesis and Characterization of Nanoparticles

Malic acid functionalized-magnetite was obtained via coprecipitation of Fe^2+^ and Fe^3+^ ions in an alkaline environment containing the functionalization agent (malic acid). MCM-41 was synthesized as previously reported in [63]. Vancomycin loading was carried out using a vacuum-assisted method. MCM-41 was left for 1 h at degassing under vacuum. This step ensures that humidity and air are removed from the porous framework of MCM-41. After degassing, the material was put in contact with a saturated aqueous vancomycin solution (125 mg/mL). Physically, the solution was adsorbed into the pores of the mesoporous material. Subsequently, the obtained loaded-material was dried at 40 °C until complete solvent evaporation. Vancomycin was loaded at 30% (*w*/*w*) in relation to the final mass of the loaded MCM-41. In order to produce the magnetic films, Fe_3_O_4_/malic and MCM-41@Van were mixed by grinding in an equal ratio by weight. 

Depending on the desired key properties, the nanoparticles were analyzed by X-ray diffraction and infrared spectroscopy in the case of magnetite, infrared and Raman spectroscopy, as well as BET analysis for mesoporous silica, both unloaded and loaded. Thermogravimetric analysis coupled with differential scanning calorimetry and dynamic light scattering were also included for all nanoparticles. 

X-ray diffraction (XRD) was performed using a PANalytical EMPYREAN diffractometer (PANalytical, Almelo, The Netherlands) equipped with a Cu Kα radiation source (Kα = 1.5406). Scans were obtained between 10–80° 2θ with an acquisition step of 0.0260.

Infrared spectra were scanned in the 4000–400 cm^−1^ range on a Nicolet iS50 FT-IR spectrometer (Waltham, MA, USA) in ATR mode. Spectra were recorded as the average of 64 scans at 4 cm^−1^ resolution. The same spectrometer was used to record the FT-Raman spectra, the instrument being equipped with a dedicated Raman hardware component with an InGaAs detector and CaF_2_ beam splitter. One hundred scans per spectrum were collected in the 4000–400 cm^−1^ range using 0.50 W laser power.

Nitrogen adsorption/desorption isotherms were measured at 77 K in the relative pressure range p/p_0_ = 0.005–1.0 using a NOVA 800 gas sorption analyzer (Anton Paar QuantaTec, Inc., Boyton Beach, FL, USA). Prior to the adsorption experiments, the materials were degassed under vacuum at 180 °C for four hours. The specific surface area was calculated using the Brunauer-Emmett-Teller (BET) equation. The amount of gas absorbed at a pressure ratio of p/p_0_ allowed the estimation of the total pore volume. The desorption branch of the isotherm was used to calculate the pore size distribution and mesopore volume using the Barrett-Joyner-Halenda (BJH) model.

For TGA-DSC, samples were weighed and placed in an alumina crucible and subjected to analysis in the temperature range 23–900 °C using a STA TG/DSC Netzch Jupiter 449 F3 instrument (Selb, Germany). 

DLS measurements were achieved after dispersing the samples in ethanol at a concentration of 0.35 mg/mL using an ultrasonic bath. Small amounts of the nanoparticle suspension were injected into the measurement cell of the DelsaMax Pro (Backman Coulter, Brea, CA, USA). Measurements were performed in triplicate for each sample.

### 4.3. Preparation and Characterization of the Vancomycin-Loaded Magnetic Alginate Film

First, a 2 wt% alginate solution (AlgNa) was prepared by dissolving the appropriate amount of sodium alginate powder in ultrapure water. The solution was left overnight under magnetic stirring so that sodium alginate was completely dissolved. Glycerol (G) was then added to the AlgNa solution (10 wt% based to the weight of sodium alginate). MCM-41@Van and the mixture of Fe_3_O_4_/malic with MCM-41@Van were further incorporated into the alginate by trituration using an agate mortar. 

Finally, the films were prepared by casting in Petri dishes, followed by overnight drying at 40 °C, and finally cross-linking with a CaCl_2_ 2% (*w*/*v*) solution, for 5 min. The cross-linking solution was prepared by dissolving CaCl_2_ in a 50/50 (*v*/*v*) ethanol/water solvent mixture. Ethanol was chosen as a co-solvent to reduce the solubility of the drug in the cross-linking solution, thereby reducing the risk of drug loss. As shown by Li et al. [64], ethanol improved the visual appearance of the alginate films and the tensile strength of the dry films with increasing ethanol concentration. Three types of films were prepared containing: (i) sodium alginate and glycerol (AG), (ii) sodium alginate-glycerol/MCM-41@Van (AGSi), and (iii) sodium alginate-glycerol/MCM-41@Van/Fe_3_O_4_/malic, (AGSiM). A summary of the mass ratios of the components for each film is given in Table 4.

In addition to the infrared spectroscopic analysis, 2D maps of the films were acquired using a Nicolet iS50R FTIR microscope (Nicolet, Waltham, MA, USA), equipped with a cooled MCT detector. 

The surface and cross-sectional morphologies of the films, as well as energy dispersive x-rays (EDX) and elemental mapping, were assessed using a scanning electron microscope (FEI Inspect F50, Eindhoven, The Netherlands) equipped with an ET detector. Samples were analyzed at 1000×, 2500× and 5000× magnification at 20.0 keV after a prior gold coating.

### 4.4. Performance of the Obtained Films

#### 4.4.1. Wound Fluid Uptake and Water Vapor Transfer Rate 

To determine the capacity for wound fluid uptake, cross-linked samples were dried at 50 °C for 12 h. After complete drying, the samples were cut into 1 cm × 1 cm areas, weighed on an analytical microbalance (Mettler Toledo XP6, Columbus, OH, SUA) and immersed in 10 mL of simulated wound fluid (SWF) prepared according to [47]. At specific time intervals, samples were removed from the medium, blotted to remove excess solvent, weighed, and then returned to the SWF until mass equilibrium was reached. The swelling ratio was then calculated using Equation (1). The tests were performed three times for each film type and the results are expressed as mean ± standard deviation.

(1) $$Swelling\;ratio=\frac{W_{w}-W_{d}}{W_{d}},$$

where W_w_ is the weight of the wet film at predetermined times and W_d_ is the weight of dry film prior to dipping in SWV fluid.

A 1 cm pathway spectrophotometric cuvette containing 4 mL of distilled water was capped with a square piece of dry film, and further sealed with Teflon tape to prevent any water vapor losses. The entire experimental set-up was weighed and placed in an incubator at 37 °C. After 24 h, the experimental set-up was weighed, and the water vapor transmission rate (WVTR) was calculated using Equation (2)

(2) $$WVTR\;\left(g/m^{2}\right)=\frac{W_{0h}-W_{24h}}{A},$$

where W_0h_ is the weight of the experimental set-up at time t = 0, W_24h_ is the weight of the experimental set-up at time t = 24 h, and A is the surface area of the film exposed to water vapor loss, expressed in m^2^.

#### 4.4.2. Vancomycin Release Behavior

Vancomycin release in aqueous medium was further investigated. Samples of 0.5 cm × 0.5 cm were cut from the films. Equal amounts of the available cut specimens were weighed and immersed in aqueous solution. At 1, 3, 9, 24 and 48 h intervals, the solutions were read spectrophotometrically at 280 nm wavelength to quantify the release of vancomycin in the medium. Quantification was possible by applying the linear equation obtained after calibration in 5–25 µg/mL range (correlation coefficient of 0.997, slope of 0.0011 and intercept of 0.0068).

#### 4.4.3. Antimicrobial Activity of Alginate-Based Films

The antimicrobial assessments were performed using *Staphylococcus aureus* ATCC 10231, *Enterococcus faecalis* ATCC 29212, *Enterococcus faecalis* VRE 2566 (vancomycin-resistant *Enterococcus*, clinical isolate) and *Candida albicans* ATCC 10231. Bacterial cell suspensions of 1.5 × 10^8^ CFU/mL (0.5 McFarland density standard) and yeast suspensions of 3 × 10^8^ CFU/mL (1 McFarland density standard) were obtained from fresh cultures on NB (for *S. aureus*), M-H (for *E. faecalis* species) and Sabouraud with agar media (for *C. albicans*).

The impact of the contaminants on the experiment was avoided by previously sterilizing all films (0.5 cm × 0.5 cm) by holding them under UV radiation for 30 min on each side. The sterility test was performed on each sample by maintaining it in NB media for 24 h at 37 °C to confirm the sterility of the tested films before the antimicrobial assay. The clarity of the broth media confirmed the sterility of the samples [65].

##### Qualitative Evaluation of the Antimicrobial Activity

The qualitative antimicrobial assay was performed using an adapted spot diffusion method [65,66]. Petri dishes containing media specific to each strain were seeded with inoculum, and films of the same size were added at equal distances. The biologically active compounds of films were used as control solutions with specific concentrations. The effect of the samples against selected pathogenic strains was evaluated after a 24 h incubation at 37 °C, when all bioactive compounds had diffused to the media surface. The sensibility of the strains was assessed by measuring the growth inhibition diameters. 

##### Quantitative Evaluation of the Anti-Adherence Capacity of the Alginate-Based Films

The anti-adherent capacity of the alginate-based films was established by determining the colony-forming units/mL (CFU/mL) using the method described in previous studies [65,67] and according to the CLSI standard [68]. The negative control was performed and considered them to be sterile media. The positive control/cell growth control was the broth media inoculated with microbial suspensions. As in the previous assay, the bioactive compounds of the films were evaluated independently at the corresponding concentrations and following the same method. Viable colony formation was expressed as the mean of the total number of colonies × 1/D (D = decimal dilution at which the total number of colonies was determined). 

Meanwhile, the release of biological compounds into the liquid media was quantitatively evaluated based on the decimal microdilution method. After incubating the samples in liquid media in the presence of the 10^7^ bacterial cells density, 30 µL of the liquid media were taken, and decimal dilutions were performed to determine CFU/mL, according to the previous method. The assays were performed in three independent experiments [65,67].

### 4.5. Statistical Analysis

Biological assessments were designed and performed in triplicate, and the results were statistically analyzed using GraphPad Prism 9.5 (GraphPad Software, San Diego, CA, USA). All experiments were performed in three independent determinations. Results are expressed as ±SD (standard deviation), and differences between alginate-based films and bioactive compounds were compared by one-way analysis of variance (one-way ANOVA), respectively Holm-Šídák’s and Dunnett’s as multiple comparisons tests. The data results were considered statistically significant if the *p*-value < 0.05.

## Figures and Tables

**Table 1:** Textural characteristics of the obtained mesoporous silicas.

Sample	Surface Area (m^2^·g^−1^)	Total Pore Volume (cm^3^·g^−1^)	Average Pore Diameter (nm)
MCM-41	1233	0.6381	3.18
MCM-41@Van	353	0.2147	2.82

**Table 2:** Tabulated TGA -DSC data of the obtained nanoparticles.

Sample	Mass Loss (%)			Thermal Effects (°C)	
	RT-200 (°C)	200–900 (°C)	Residual Mass	Endothermic	Exothermic
Fe_3_O_4_/malic	9.19	7.35	83.46	93.5	215.0
					257.0
					486.9
MCM-41	1.35	6.57	92.17	55.1	-
MCM-41@Van	9.02	30.24	60.74	84.0	352.0
					540.3

**Table 3:** Growth inhibition zone diameters (GIZD).

Strains	GIZD (mm)							
	AG	AGSi	AGSiM	MCM-41	MCM-41@Van	Van	Malic Acid	Fe_3_O_4_@ Malic
*Staphylococcus aureus* ATCC 10231	18.00 ± 1.00	27.50 ± 1.32	29.83 ± 0.29	2.00 ± 0.05	29.73 ± 0.64	20.10 ± 0.36	16.20 ± 0.52	9.93 ± 0.70
*Enterococcus faecalis* ATCC 29212	4.83 ± 0.76	29.60 ± 0.52	34.00 ± 1.00	1.13 ± 0.23	39.90 ± 0.85	20.23 ± 0.32	9.00 ± 1.00	10.03 ± 0.45
*Enterococcus faecalis* VRE 2566	14.93 ± 0.30	23.80 ± 0.72	32.67 ± 0.57	2.13 ± 0.23	35.67 ± 0.57	19.50 ± 0.50	9.50 ± 0.50	10.87 ± 0.23
*Candida albicans* ATTC 10231	1.07 ± 0.11	10.23 ± 0.2	11.97 ± 0.45	2.10 ± 0.17	2.76 ± 0.66	1.56 ± 0.32	1.16 ± 0.11	14.27 ± 1.27

The significant impact of the samples on each microbial strain was statistically analyzed by one-way ANOVA and Holm-Šídák’s multiple comparisons post hoc test. The resulting data were statistically significant (*p* < 0.0001).

**Table 4:** Summary of the components ratio and the corresponding sample labeling.

Component	AG	AGSi	AGSiM
Sodium alginate/glycerol	1.0	0.75	0.50
MCM-41@Van	-	0.25	0.25
Fe_3_O_4_/malic acid	-	-	0.25

## Data Availability

Not applicable.
